# Establishing a Multidisciplinary Head and Neck Clinical Pathway: An Implementation Evaluation and Audit of Dysphagia-Related Services and Outcomes

**DOI:** 10.1007/s00455-018-9917-4

**Published:** 2018-06-19

**Authors:** Barbara Pisano Messing, Elizabeth C. Ward, Cathy Lazarus, Keri Ryniak, Melissa Kim, Jessica Silinonte, Dorothy Gold, Carol B. Thompson, Karen T. Pitman, Ray Blanco, Ryan Sobel, Karen Harrer, Karen Ulmer, Geoffrey Neuner, Kruti Patel, Mei Tang, Gregory Lee

**Affiliations:** 1 Greater Baltimore Medical Center, The Milton J Dance, Jr. Head and Neck Center, Johns Hopkins Head & Neck Surgery, Johns Hopkins Voice Center, Baltimore, MD USA; 2 School of Health and Rehabilitation Sciences, The University of Queensland, Brisbane, QLD Australia; 3 Centre for Functioning and Health Research, Metro South Hospital and Health Service, Queensland Government, Brisbane, QLD Australia; 4 Department of Otolaryngology-Head & Neck Surgery, Icahn School of Medicine at Mount Sinai, Thyroid Head and Neck Research Center, Thyroid Head and Neck Cancer (THANC) Foundation, Mount Sinai Beth Israel, New York, NY USA; 5 Johns Hopkins Biostatistics Center, Johns Hopkins Bloomberg School of Public Health, Baltimore, MD USA; 6 Department of Otolaryngology-Head and Neck Surgery, Johns Hopkins Medical Institutions, Baltimore, MD USA; 7 Greater Baltimore Medical Center, The Milton J. Dance, Jr. Head & Neck Center, 6569 N. Charles Street, PPW Suite 401, Baltimore, MD 21204 USA

**Keywords:** Head and neck cancer, Clinical pathway, Deglutition, Deglutition disorders, Dysphagia, Nutrition, Oral intake, Implementation

## Abstract

Head and neck cancer (HNC) guidelines recommend regular multidisciplinary team (MDT) monitoring and early intervention to optimize dysphagia outcomes; however, many factors affect the ability to achieve these goals. The aims of this study were to explore the barriers/facilitators to establishing and sustaining a MDT HNC care pathway and to examine the dysphagia-related speech-language pathology (SLP) and dietetic components of the pathway. Using the Consolidated Framework for Implementation Research (CFIR), a mixed methods study design was used to evaluate an established MDT HNC pathway. Ten MDT members provided perceptions of facilitators/barriers to implementing and sustaining the pathway. Patients attending the SLP and dietetic components of the pathway who commenced treatment between 2013 and 2014 (*n *= 63) were audited for attendance, outcome data collected per visit, and swallowing outcomes to 24-month post-treatment. Dysphagia outcomes were compared to a published cohort who had received intensive prophylactic dysphagia management. Multiple CFIR constructs were identified as critical to implementing and sustaining the pathway. Complexity was a barrier. Patient attendance was excellent during treatment, with low rates of non-compliance (< 15%) to 24 months. Collection of clinician/patient outcome tools was good during treatment, but lower post-treatment. Dysphagia outcomes were good and comparable to prior published data. The pathway provided patients with access to regular supportive care and provided staff opportunities to provide early and ongoing dysphagia monitoring and management. However, implementing and sustaining a HNC pathway is complex, requiring significant staff resources, financial investment, and perseverance. Regular audits are necessary to monitor the quality of the pathway.

## Introduction

The nature of head and neck cancer (HNC) presents a complex and challenging environment for professionals working with this population. The disease process, comorbidities, and a myriad of psychosocial factors necessitate the optimization of patient care with a systematic approach. Current research has established that HNC care delivered through an integrated Multidisciplinary Team (MDT) approach results in improved patient outcomes and better survival rates [1–3]. Delivery of MDT services through a coordinated head and neck clinical pathway (HNCP) is also recognized to maximize results, increase efficiency in care delivery, reduce costs, shorten the length of hospital stay, and improve overall patient outcomes [4–7].

Clinical pathways in HNC care strive to provide evidence-based algorithms by organizing patient care in a coordinated and systematic MDT approach [4]. International cancer care guidelines advocate that HNC services be provided by the MDT housed in an established, patient-centered head-and-neck oncology center with a dedicated team. This team includes specialized medical staff (e.g., Head and Neck Surgeon, Medical Oncologist, Radiation Oncologist), nursing, speech-language pathologists, dietitians, social workers, and administrative professionals (e.g., systems analyst, clinical research coordinator) [8–10]. Routine and ongoing patient monitoring by the MDT pre, during, and post-treatment is also advocated in the recent literature [8, 9]. However, implementing a true MDT clinical pathway in today’s complex healthcare environment is fraught with roadblocks and pitfalls. Barriers to adequate treatment are not an isolated problem, but multifaceted. Developing a MDT care pathway requires a significant amount of resources initially, as well as on an ongoing basis, to achieve sustainability.

One of the significant negative impacts of HNC and its treatment is the decline of swallow function combined with inadequate nutritional intake. Loss of swallow function and reduced nutritional status can be present from the time of initial diagnosis, become exacerbated during treatment due to related toxicities, and persist long-term for many patients. Because of this, it is recognized in practice guidelines that swallowing and nutritional status should be part of routine assessment pre-treatment and continue to be monitored during and post-treatment [8]. Furthermore, the importance of early and active intervention to help maximize a patient’s functional swallowing outcomes is becoming well-recognized. Recent evidence supports that providing prophylactic exercises during and post-treatment may improve a patient’s swallow function, which impacts nutritional status and overall quality of life during treatment and long-term post-treatment [11–21]. With less functional decline, patients were able to return to an oral diet sooner, leading to less weight loss and shorter enteric tube duration [13, 15, 17, 21, 22]. To this end, routine swallowing and nutritional monitoring, from the point of diagnosis, as well as early swallow intervention should be part of any larger MDT care pathway for this population. However, despite acknowledging this evidence, not all clinical services are currently able to deliver an integrated MDT model of care, particularly for comprehensive management of deglutition disorders [23, 24].

The overall purpose of the current study was to conduct a service implementation evaluation of an integrated MDT HNC care pathway developed from best practice research evidence, incorporating speech-language pathology (SLP) and dietetic services to optimize treatment of swallowing disorders. The specific aims of this study were to explore the barriers and facilitators to establishing and sustaining this MDT HNC care pathway and then to examine the outcomes of the SLP and dietetic components of the larger MDT HNC care pathway. Within the field of implementation science, this type of formative evaluation is considered essential to promoting sustainability of an “intervention” within the service and can assist uptake of any “intervention” in other contexts. This research is intended to provide insights and considerations that may assist future services to establish systems that optimize deglutition within the MDT HNC care pathways.

## Methods

**Fig. 1:**
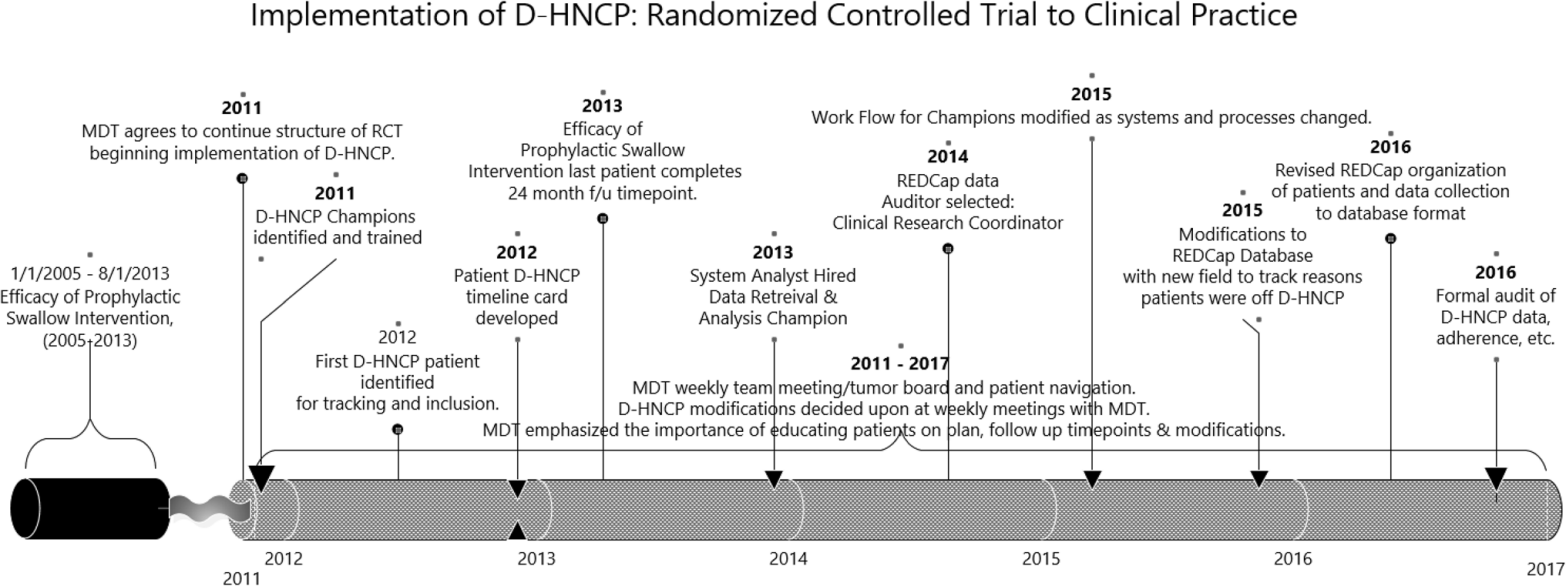
Dance Head and Neck Clinical Pathway (D-HNCP) implementation timeline

The Dance Head and Neck Clinical Pathway (D-HNCP) was first established in 2011 within the Milton J. Dance, Jr. Head and Neck Center (Dance Center), in Baltimore, MD, USA. The Dance Center is a tertiary cancer service providing HNC services to approximately 400 newly diagnosed cancer patients annually. The D-HNCP was developed following the implementation of a randomized controlled trial (RCT) between 2003 and 2013 within the same service that had introduced a structured, protocol-based method of navigating and coordinating HNC patient care, collecting routine patient-reported outcomes (PROs) and clinician-reported outcomes (CROs), and providing prophylactic swallowing exercises/therapy to HNC patients with stage 3–4 squamous cell carcinoma [25]. In 2011, toward the end of the RCT, positive clinician and patient experiences facilitated the transition from simply following the research protocol for a specific cohort, to adopting the practices more broadly as part of a routine clinical pathway of care for all patients. The first patient recruitment/data collection to the D-HNCP commenced in June 2012 (Fig. 1).

### The Dance Head and Neck Clinical Pathway (D-HNCP)

**Fig. 2:**
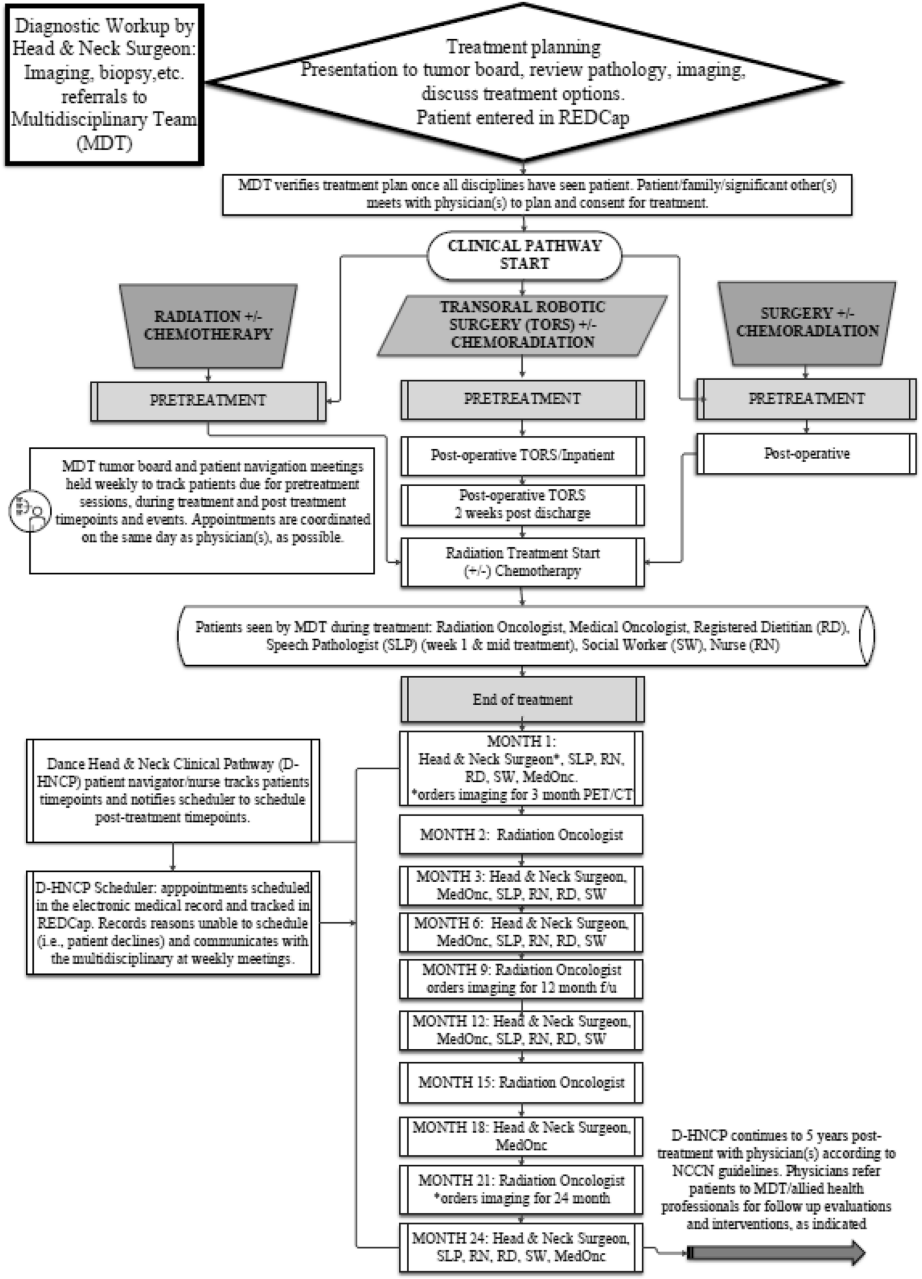
Overview of Dance Head and Neck Clinical Pathway (D-HNCP) key time points from baseline to post-treatment for multidisciplinary team (MDT). [REDCap (Research Electronic Data Capture), NCCN National Comprehensive Cancer Network^®^ (NCCN^®^)]

The D-HNCP was developed to ensure patients were followed regularly by an MDT of professionals, through scheduled coordinated appointments, from initial diagnosis up through 5 years post-treatment (Fig. 2). Patient groups eligible for management within the D-HNCP include all patients attending the service planned for curative treatment for HNC including those scheduled to receive: chemoradiation (CRT); Transoral Robotic Surgery ± chemotherapy/radiation therapy; or surgery ± chemotherapy/radiation therapy. Exceptions include patients with thyroid or skin cancer, small tumors (e.g., T1N0M0), those scheduled for total laryngectomy, patients referred to the service following treatment elsewhere, or any patient with recurrent disease. These excluded patients received alternate care plans.

The team of professionals involved in the D-HNCP includes head and neck surgeons, medical oncologists, radiation oncologists, dentists, speech-language pathologists, oncology dietitians, nurse specialists, oncology social workers, physical therapists, and occupational therapists. Each member of the MDT has designated information and outcome measures that are collected on patients at key timepoints on the D-HNCP. Within the D-HNCP, routine CROs and PROs are collected to monitor patient performance. The CROs and PROs specific to management of deglutition disorders by SLP and dietetics services are detailed in Table 1 [25–31].

**Table 1:** Routine data collection and time points for speech pathology and dietetic contact within the D-HNCP

Outcome Measures	Pre-treatment	Week 1 CRT^a^	Week 3/4 CRT	Months post CRT				
				1	3	6	12	24
Dietetics								
Weight	x	x	x	x	x	x	x	x
Presence and use of non-oral feeding	x	x	x	x	x	x	x	x
Speech language pathology								
Oral motor examination^b^	x	x	x		x			x
Incisal opening^c^	x	x	x		x	x	x	x
Maximal lingual pressure^d^	x	x	x		x			x
Performance status scale (PSS)^e^	x	x	x	x	x	x	x	x
Functional oral intake score (FOIS)^f^	x	x	x		x			x
Modified barium swallow	x				x			x
EAT-10^g^	x	x	x	x	x	x	x	x
EORTC C30 and HN35^h^	x				x			x
Sydney swallow questionnaire (SSQ)^i^	x	x	x		x			x

Exceptions to the Dance Head & Neck Clinical Pathway (D-HNCP): (a) patients undergoing extensive reconstructions do not complete a pre-treatment MBS, (b) patients receiving transoral robotic surgery (TORS) complete 2 additional contacts at day 1–3 and 2 weeks post TORS during which all assessments are completed except quality of life and an endoscopic assessment is completed instead of a MBS

^a^Chemoradiation treatment (CRT)

^b^Oral Motor Examination: as measured using the Milton J., Dance, Jr. Head & Neck Center Rating of Oral Motor Tool [25]

^c^Incisal opening: as measured using the Therabite^®^ measuring tool

^d^IOPI: as measured using the Iowa oral pressure instrument (IOPI)

^e^Performance status scale (PSS) [26]

^f^Functional oral intake (FOIS) [27]

^g^EAT-10 [28]: patient-reported swallow outcome

^h^European Organization for Research and Treatment of Cancer (EORTC QLQ-C30) and the Head and Neck Cancer Quality of Life module (EORTC QLQ-HN35) [29, 30]

^i^Sydney Swallow Questionnaire (SSQ) [31]

Within the D-HNCP, patients receive regular SLP and dietetic services in coordination with other MDT appointments, starting at pre-treatment and continuing up to 24-month post-treatment. These appointments are designed to maximize nutrition, swallowing and quality-of-life through close monitoring, and to facilitate initiation of prophylactic swallow-therapy interventions and delivery of post-treatment management of deglutition disorders. Details of the timing of SLP and dietetic appointments and the assessments collected as part of the D-HNCP are outlined in Table 1. Coordinating interdisciplinary scheduling of patient appointments with MDT’s availability creates significant challenges requiring daily monitoring to ensure accuracy. The complexity of scheduling the D-HNCP required the leader of the D-HNCP to identify champions who would be responsible for overseeing and correcting errors in scheduling.

Regarding swallow rehabilitation, during the week 1 and mid-radiation treatment appointments, the SLP instructs patients on prophylactic swallowing exercises. These exercises included lingual strengthening, Masako maneuver, effortful or supraglottic swallow, Mendelson maneuver and the Shaker exercise. A TheraBite^®^ Jaw Motion Rehabilitation System™ (www.atosmedical.com) is dispensed and instruction provided when a patient is identified as having reduced incisal opening as measured by the TheraBite^®^ measuring device to be less than 40 mm. Expiratory muscle strength training (EMST) devices are utilized as part of the treatment program to improve airway clearance and airway protection in patients who are at risk for penetration and aspiration [32–36]. Patients were encouraged to complete their prescribed exercises twice daily, 6 days a week; however, the severity of acute treatment toxicities influenced adherence. Post CRT appointments involved instruction in individualized swallowing therapy programs, as determined by the nature and severity of presenting deficits.

**Fig. 3:**
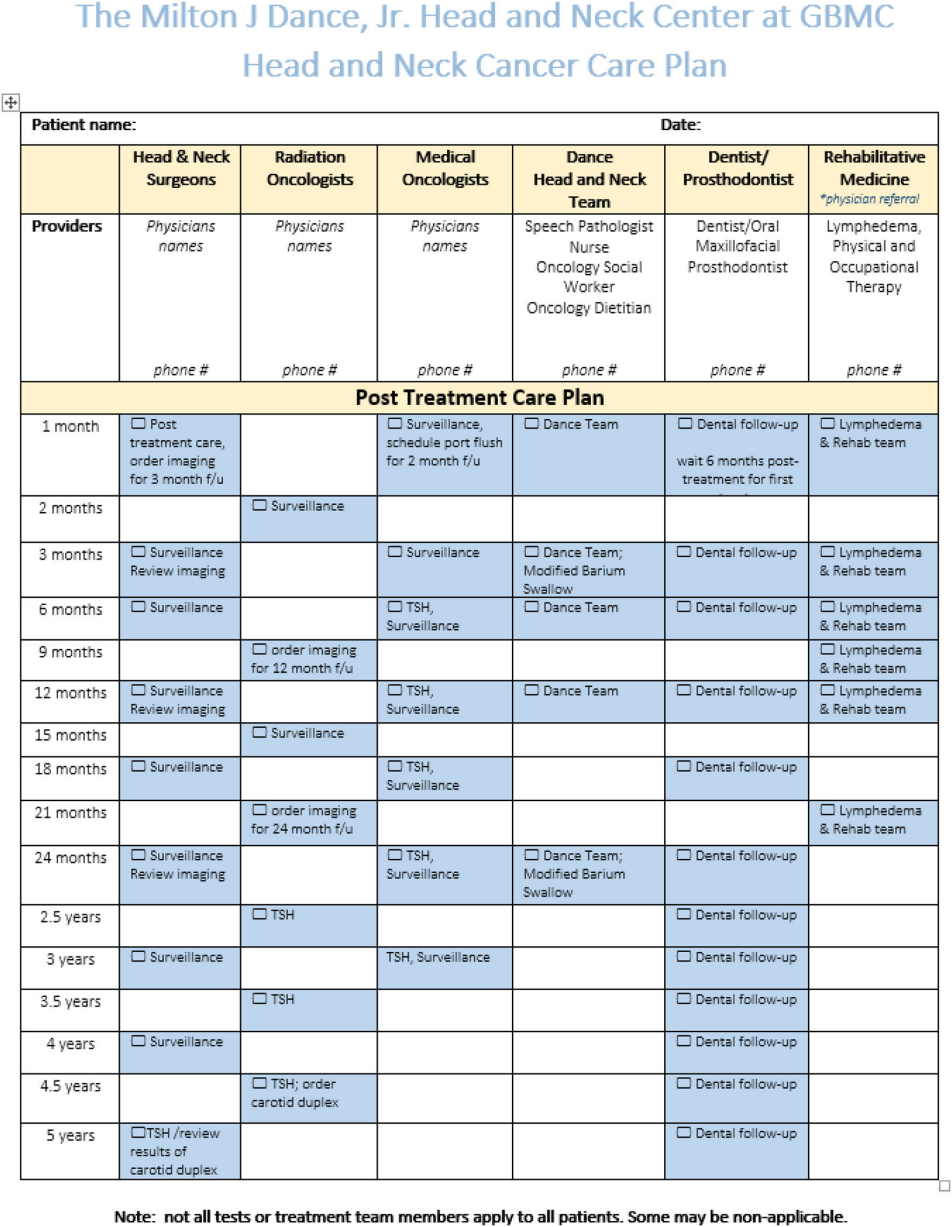
Multidisciplinary team patient care plan. *GBMC* Greater Baltimore Medical Center

The D-HNCP “patient care plan form” is provided to all patients to educate them on the clinical pathway and appointment timepoints post-surgery and post-radiation (± chemotherapy). An example of this is provided in Fig. 3. The patient care plan form also briefly describes the purpose of the pathway to ensure that patients understand how they will be followed post-treatment by the MDT. Appointment reminders (i.e., phone calls, mail, email) are provided to keep patients on track, to help patients understand their treatment plan and to improve communication between the MDT members and the patient/family.

All D-HNCP data from each appointment are collected, entered, and managed using the REDCap (Research Electronic Data Capture) [37]. REDCap is a secure, web-based application designed to support data capture for research studies, providing (1) an intuitive interface for validated data entry; (2) audit trails for tracking data manipulation and export procedures; (3) automated export procedures for data downloads to common statistical packages, and (4) procedures for importing data from external sources. The REDCap system facilitates the coordination of appointments and sharing of clinical data across the D-HNCP. Performance improvement activities are an integral component of the D-HNCP and are completed through REDCap. Periodically, performance improvement and audits have followed the Plan-Do-Study-Act and Smarter Goals formats to examine how to improve scheduling issues, reasons for visits and timeliness of documentation [38].

### Implementation Evaluation

The current health service incorporates a mixed-methods approach to evaluate the implementation of the multidisciplinary D-HNCP and to explore the SLP and dietetic components and outcomes relating to the management of deglutition disorders. A survey with key members of the MDT was conducted as part of this study to examine barriers and facilitators to implementing the D-HNCP. A service audit of a 2-year cohort of patients was also conducted, from the point of initial contact to 24-month post-treatment, to examine adherence to the pathway and the collection of CROs/PROs. Swallowing outcomes up to 24-month post-treatment were also collected for a subset of this cohort and compared to a published research cohort [25]. This research was conducted under the Institutional Research Board (IRB) approval # 516223.

### Guided Survey with MDT Members

Exploration of influencing factors for establishing and sustaining the D-HNCP was conducted using a guided survey format with key stakeholders involved in the D-HNCP. Only those MDT members who had at least five or more years of experience with the pathway were eligible for participation. The MDT clinical staff have worked at the Dance Center for an average of 12 years (range 5–28 years), and hence, most of the core team was eligible for participation. Surveys were conducted with groups of up to three participants based on scheduling and availability.

The Consolidated Framework for Implementation Research (CFIR) [39], an established framework for evaluating health service interventions, was used to develop the structured guide for the survey sessions. The CFIR tool was developed following synthesis of multiple existing implementation frameworks and provides a structure to support and guide formative evaluations of health service implementation. The tool consists of five key domains, and within each of these five domains are constructs that may influence implementation (Table 2) [39]. Of the five domains, only four domains (*Intervention Characteristics, Inner Setting, Outer Setting,* and *Process*) were explored in this study based on their relevance to the study’s purpose, as per the “menu of constructs” approach used by Damschroder and Lowery [40]. Within these domains, all constructs were examined except for ‘cosmopolitanism’ (from *Domain: Outer Setting*), and ‘key stakeholders,’ and ‘innovation participants’ (from *Domain: Process*). ‘Cosmopolitanism’ (the degree the service is networked to other external organizations) was not examined as the Dance Center and services are housed within a free-standing hospital. As ‘key stakeholders’ were the participants in the interviews, and the ‘innovation participants’ (i.e., patients receiving the pathway of care) were not involved in the pathway evaluation, the influence of those specific constructs was also not able to be examined.

**Table 2:** Perceived influence of CFIR domains and constructs for establishing and sustainability of the D-HNCP

	Establishing the D-HNCP (*n* = 10)			Sustainability of the D-HNCP (*n* = 10)		
	Facilitator %	Barrier %	Low-impact/non-applicable %	Facilitator %	Barrier %	Low-impact/non-applicable%
Intervention characteristics						
Intervention source	90	0	10	60	0	40
Evidence strength and quality	80	0	20	90	0	10
Relative advantage	90	0	10	70	0	30
Adaptability	100	0	0	80	10	10
Trial ability	90	0	10	90	0	10
Complexity	30	60	10	10	60	30
Design quality and packaging	40	30	30	40	30	30
Cost	20	30	50	0	30	70
Outer setting						
Patient needs and resources	90	10	0	80	20	0
Peer pressure	10	20	70	20	10	70
External policy and incentives	10	10	80	10	20	70
Inner setting						
Structural characteristics	70	0	30	70	0	30
Networks and communications	90	0	10	80	10	10
Culture	90	0	10	90	0	10
Implementation climate	90	10	0	80	20	0
Tension for change	0	20	80	0	40	60
Compatibility	80	10	10	90	10	0
Relative priority	80	10	10	90	10	0
Organizational incentives and rewards	10	10	80	10	10	80
Goals and feedback	100	0	0	90	0	10
Learning climate	90	10	0	90	10	0
Readiness for implementation	100	0	0	100	0	0
Leadership engagement	70	10	20	80	10	10
Available resources	100	0	0	90	10	0
Access to knowledge and information	80	20	0	90	10	0
Process						
Planning	100	0	0	90	10	0
Engaging	80	20	0	70	30	0
Opinion leaders	100	0	0	70	20	10
Formally appointed internal implementation leaders	70	20	10	70	20	10
Overall championing	80	10	10	70	30	0
External change agents	40	0	60	40	0	60

Bold = 60% or more identified a construct as a facilitator or barrier

Using the online resource Consolidated Framework for Implementation Research Constructs [41], key statements were generated and proposed to the stakeholders. They were then asked to consider and indicate on the data collection forms if the construct was either a facilitator (positive influence), a barrier (negative influence) or a factor with little impact (neutral influence/not applicable) on both the (a) initial establishment and (b) the sustainability of the D-HNCP. Questions and clarification of any construct were encouraged. However, individuals completed their ratings of each construct independently on a de-identified paper form, which was submitted after the session to a separate staff member not involved in the survey sessions. Only once surveys from all MDT members were completed, were the full set of responses provided to the research team for analysis to preserve anonymity. If 60% or more of the staff identified a construct as a facilitator/barrier, it was considered in the analysis.

### Service Audit and Outcomes

The service audit examined the SLP and dietetic patient and clinical reported outcomes (CRO’s, PRO’s). Data used in the audit were extracted from the REDCap database by the service data manager. For the audit, any patient who commenced HNC treatment within the D-HNCP during 2013 or 2014 and met the inclusion/exclusion criteria were included, providing two years of new admissions. All patients in these two years were followed from baseline to up 24-months post-treatment.

For each included patient, information regarding attendance for all SLP and dietetic sessions to 24-month post-treatment was collected. A patient was determined to be non-adherent at a time point if they failed to respond to follow-up appointment requests or were scheduled and failed to show for their appointment with the allied health professionals of the MDT, even if they attended the physician appointments (e.g., head and neck surgeon, radiation oncologist, medical oncologist). The completion of the CROs/PROs scheduled for collection by clinicians at each time point was also audited. CRO/PRO data collection was examined for each participant at each time point, as percent of outcome measures collected by the SLP and/or RD.

Swallowing outcome data from the pre-treatment, 3, 6, 12, and 24-month assessments were audited for the smaller subset of D-HNCP patients who received CRT. This specific subset of patients was selected so that their data could be directly compared to an existing cohort of chemoradiation patients from the same service, reported in a previously published manuscript [25]. Specifically, the existing cohort’s data were used as a comparison service model, since they had received active routine monitoring during and post-treatment and had received active prophylactic swallowing intervention, similar to the D-HNCP patients. Parameters collected for both cohorts were detailed in a previously published manuscript [25]. These parameters included weight, percutaneous gastrostomy tube (PEG) in situ, oromotor assessment, size of incisal opening, Functional Oral Intake Scale (FOIS) [27] for present diet level of foods and fluids, the presence of penetration–aspiration on thin liquids as scored on the penetration–aspiration scale [42], and number of oral, pharyngeal and esophageal problems determined during a baseline and 3-month post-treatment Modified Barium Swallow Study (MBS) and coded as a binary measure of normal versus abnormal [25]. Statistical comparisons between the two groups, at the baseline, 3, 6, 12, and 24-month post-treatment timepoints in the pathway were performed using Fisher’s exact test or *t* test (with unequal variances) depending on the data. Statistical significance was set at *p* < 0.05 and was not adjusted for multiple comparisons.

## Results

### Guided Survey Assessment

Ten MDT staff completed the guided surveys including: Head and Neck Surgeon (*n* = 1), SLPs (*n* = 3), social workers (*n* = 2), dietitian (*n* = 1), nurse (*n* = 1), clinical research coordinator (*n* = 1), and the senior medical secretary responsible for scheduling D-HNCP patient appointments (*n* = 1). Summary of the percentage of all staff who perceived each CFIR construct as a barrier/facilitator for implementation and sustainability of the D-HNCP are detailed in Table 2.

Multiple constructs within the *Intervention Characteristics* domain were important for establishing the service (Table 2). Over 90% of MDT participants acknowledged the benefits of having trialed the pathway through the structure and design of the previous research trial and the existing evidence-base that supported the pathway’s model structure. Within the *Outer Setting,* ‘patient needs and resources’ was viewed by 90% as a facilitating factor for implementation. Most constructs in the *Inner Setting* were reported to be facilitators, highlighting the positive benefits of the ‘implementation climate’ (i.e., staff’s attitudes, team factors), ‘system structure,’ and ‘culture.’ Within the final *Process* domain, multiple constructs relating to team engagement and leadership were considered key to supporting implementation. When asked to reflect on constructs influencing ongoing service sustainability, respondents identified similar key constructs as facilitators of establishing the D-HNCP (Table 2). Only the ‘complexity’ of the pathway was considered a barrier to implementation and sustainability. Of note, the ‘cost’ was considered a low-impact factor for both implementation and sustainability.

### Service Audit

During the designated period, a total of 63 eligible patients commenced management in the D-HNCP (37 in 2013; 26 in 2014) and were included in the service audit. Demographics of these 63 patients are described in Table 3 [25]. Patients in the D-HNCP were primarily males (84%), a median age of 65 (range 49–81) with biopsy-proven squamous cell carcinoma T2 tumors (overall stage 4, 88.4%) of the oropharynx (76.7%).

**Table 3:** Patient and tumor characteristics of total D-HNCP cohort, the subgroup of chemoradiation therapy (CRT) patients in the D-HNCP and the reference research cohort [25]

Characteristics	Total D-HNCP cohort *n* = 63	CRT cohort only		
		D-HNCP CRT cohort *n* = 43	Reference research cohort *n* = 30	*p* value
Age at enrollment^a^				
Median (IQR)	62 (13)	65 (10)	55.5 (11)	< 0.001
Min–max (range)	44–81 (37)	49–81 (32)	44–78 (34)	
Gender^b^: male, *n * (%)	64 (87.0%)	36 (83.7%)	28 (93.3%)	0.195
Tumor histology				
Squamous cell carcinoma		43 (100%)	30 (100%)	n/a
Tumor location^c^, *n * (%)				0.487
Larynx, hypopharynx, nasopharynx	14 (19.2%)	8 (18.6%)	6 (20.0%)	
Oropharynx	57 (78.1%)	33 (76.7%)	24 (80.0%)	
Unknown primary	2 (2.7%)	2 (4.7%)	0	
Overall stage^c^				0.025
2	2 (2.7%)	0	2 (6.7%)	
3	14 (19.2%)	5 (11.6%)	9 (30.0%)	
4, 4a, 4b	57 (78.1%)	38 (88.4%)	19 (63.3%)	
T stage^c^				0.046
0–1	13 (17.8%)	9 (20.9%)	4 (13.3%)	
2	33 (45.2%)	22 (51.2%)	11 (36.7%)	
3	17 (23.3%)	5 (11.6%)	12 (40.0%)	
4	10 (13.7%)	7 (16.3%)	3 (10.0%)	
N stage^c^				0.227
0–1	19 (26.0%)	8 (18.6%)	11 (36.7%)	
2a, 2b	37 (50.7%)	25 (58.1%)	12 (40.0%)	
2c	14 (19.2%)	9 (20.9%)	5 (16.7%)	
3, 3b	3 (4.1%)	1 (2.3%)	2 (6.7%)	
M stage				n/a
0	73 (100%)	43 (100%)	30 (100%)	
1	0	0	0	

Bolded values indicate *p* < 0.05

IQR interquartile range

^a^Mann–Whitney test (non-parametric)

^b^Fisher’s exact test

^c^Pearson Chi-Square test

### Attendance and PRO/CRO Reporting

**Fig. 4:**
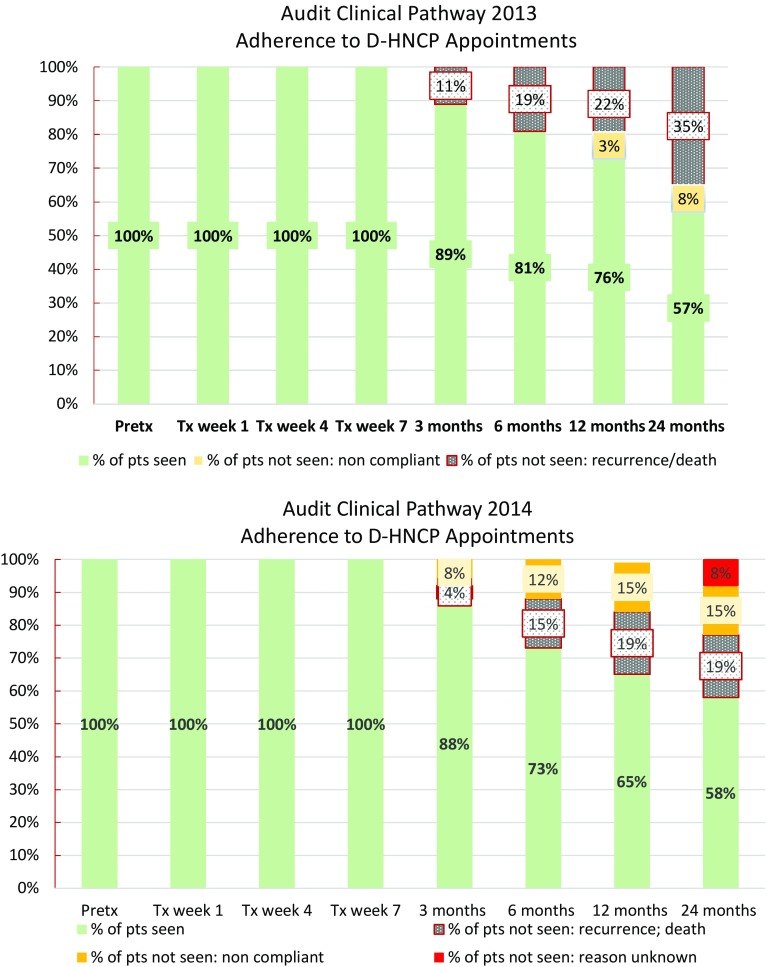
Audit of the Dance Head and Neck Clinical Pathway (D-HNCP): adherence 2013–2014 during and post-treatment

**Fig. 5:**
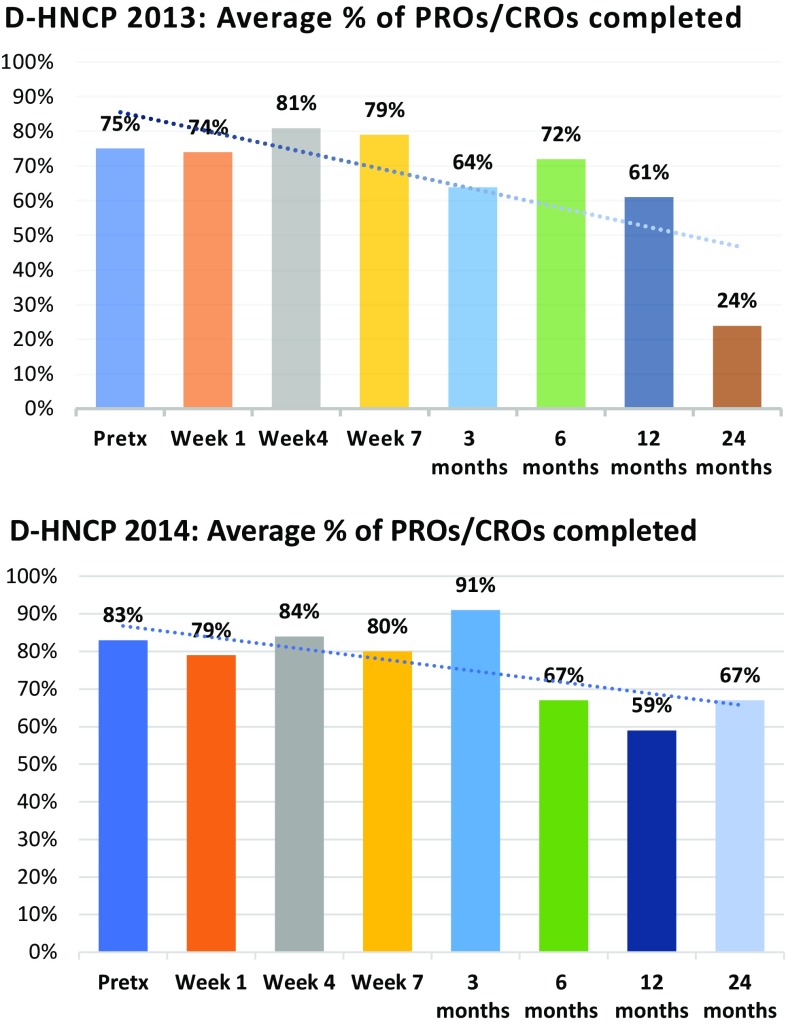
Audit of the Dance Head and Neck Clinical Pathway (D-HNCP) cohort: completion of 2013–2014 PROs/CROs during and post-treatment

Of the 37 patients admitted to the D-HNCP in 2013, adherence with attending scheduled SLP and dietetic appointments was 100% before and during treatment (Fig. 4). From 3 months on, there was attrition of 11–35% due to tumor recurrence, patient relocation or transfer for treatment, or death. Issues of non-adherence with appointments, however, were only 2% (of *n* = 29) and 8% (of *n* = 24) of cases at 12 and 24 months, respectively. For patients who commenced treatment in 2014, a similar pattern was observed (Fig. 4), though rates of non-adherence due to a patient’s failure to keep the appointments, excluding patients with a recurrence, were slightly higher (8–15%). Average clinician adherence to the collection/reporting of outcome measures for this cohort was relatively good pre-treatment and during treatment (range 74–81% in 2013; 79–84% in 2014) (Fig. 5). However, there was a gradual decline in outcome data collection at 3, 6, and 12-month post-treatment across both years of patient groups (range 61–72% 2013 group; 59–67% 2014 group). Highs and lows were seen at 3-month post-treatment with 64% in the 2013 group compared to 91% in 2014 group. At 24-month post-treatment, there was also variability in data collection between the 2013 and 2014 groups at 24 and 67%, respectively (Fig. 5).

### Swallowing Outcomes: Comparison Between Cohorts

Swallowing outcomes of only those patients in the D-HNCP who received CRT (*n* = 43) were compared to outcomes from the CRT cohort reported previously from research at this same site [25]. Demographics of this subgroup and the reference research cohort are provided in Table 3. Comparisons between the two cohorts indicated no statistically significant differences on gender, tumor site, and pathology (Table 3). Statistically significant differences indicated that the D-HNCP cohort was older by almost ten years and contained a slightly higher proportion of patients with Stage 4 tumors.

The swallowing outcomes from these two CRT cohorts are provided in Tables 4 and 5. There were no statistically significant differences with two exceptions. The D-HNCP cohort had a higher percentage of patients with swallowing problems based on the 1–5 scores on the FOIS (14% vs. 0%, *p* = 0.04) at baseline, and a lower percentage for number of patients with a PEG in situ at 3 months (57% vs. 85%, *p* = 0.02) compared to the research cohort. Although slightly higher percentages of oral and pharyngeal problems were found on the MBS Study in the D-HNCP cohort at baseline, the outcomes between the two cohorts were not statistically different (Tables 4, 5 [25, 42]).

**Table 4:** Analysis^a^ of swallowing outcomes of the chemoradiation therapy (CRT) patients in the D-HNCP cohort (*n * = 43) and the reference research sample [25] (*n * = 30)

Parameter	Baseline/pre-treatment			3-month post CRT			6-month post-CRT			12-month post-CRT			24-month post-CRT		
	Reference research cohort	D-HNCP CRT Cohort	*p*	Reference research cohort	D-HNCP CRT Cohort	*p*	Reference research cohort	D-HNCP CRT Cohort	*p*	Reference research cohort	D-HNCP CRT Cohort	*p*	Reference research cohort	D-HNCP CRT Cohort	*p*
Oral/non-oral Intake															
FOIS 1–5^b^	0%	14%	0.04	50%	43%	0.60	27%	21%	0.75	12%	21%	0.48	0%	5%	1.00
PEG in situ^c^	100%	91%	0.14	85%	57%	0.02	38%	16%	0.08	14%	3%	0.20	3%	0%	1.00
Food category 2–5^d^	13%	19%	0.75	68%	46%	0.12	39%	33%	0.78	24%	17%	0.73	14%	10%	1.00
Fluid category 2–5^e^	0%	0%	n/a	9%	0%	0.36	9%	0%	0.16	4%	0%	1.00	0%	0%	n/a
Oromotor and toxicities															
Oromotor assessment^f^	11%	17%	0.72	0%	21%	0.06	5%	23%	0.13	8%	n/a	n/a	9%	0%	n/a
Incisal opening^g^	50.7 (5.9)	48.0 (8.8)	0.13	43.2 (8.5)	48.0 (7.1)	0.05	43.9 (8.5)	42.8 (12.6)	0.75	46.7 (7.4)	44.6 (6.9)	0.36	48.6 (8.8)	48.5 (7.4)	0.97
Weight (pounds)	202.6 (44.4)	190.3 (41)	0.24	173.3 (19.7)	170.6 (36.7)	0.77	167.4 (24.0)	174.2 (35.2)	0.62	176.6 (25.4)	181.8 (33.1)	0.52	179.8 (26.3)	192.5 (32.9)	0.20

This table percentage for binary outcomes and mean(SD) for continuous outcomes. Bolded values indicate *p* < 0.05

n/a non/applicable

^a^Fisher’s exact test was used to compare the percentages at each time point (p)

^b^Functional oral intake scale (FOIS) [27] expressed as proportion of group receiving a rating of 1-5 indicating more impaired and more restricted diet level

^c^Proportion of group with a percutaneous gastrostomy (PEG) in situ

^d^Proportion of patients managing food consistencies other than normal

^e^Proportion of patients managing fluid consistencies other than normal

^f^Percentage of patients with total score ≤ 65 indicating impaired function

^g^Incisal opening actual score measured with Therabite^®^ measuring device in millimeter measurement. Normal ≥ 40

**Table 5:** Analysis of Swallow physiology at baseline and 3-month post-chemoradiation therapy patients in the D-HNCP cohort (*n* = 43) and the reference research sample (*n* = 30) [25]

Modified barium swallow parameter	Baseline/pre-treatment			3 months post-treatment		
	Research cohort (%)	D-HNCP cohort (%)	*p*	Research cohort (%)	D-HNCP cohort (%)	*p*
Oral phase impairments^a^	10	19	0.48	10	6	1.0
Pharyngeal phase impairments^a^	41	61	0.20	42	50	0.74
Esophageal phase impairments^a^	36	31	0.78	47	38	0.73
PAS penetration 2–5^b^	10	6	0.67	22	6	0.34
PAS aspiration 6–8^c^	7	6	1.0	0	19	0.09

Fisher’s exact test was used to compare the percentages at each time point (p)

^a^Oral, pharyngeal, and esophageal phase impairments. Percentage of patients with at least one problem

^b^Penetration–aspiration scale (PAS) [42] penetration: percentage of patients with penetration score of 2–5

^c^Penetration–aspiration scale (PAS) [42] aspiration: percentage of patients with aspiration score of 6–8, aspirator on thin liquids only

## Discussion

While evidence supports the importance of regular and ongoing interventions in optimizing patient outcomes, the reality is that many patients continue to face significant challenges accessing interdisciplinary, coordinated care to manage their swallowing/nutritional issues during and post HNC care [11, 23, 43, 44]. In the current study, however, a pathway of care was successfully implemented, and the results of the audit confirmed that regular and ongoing services were provided and attended by most patients, with positive long-term swallowing outcomes achieved by most as measured by PROs and CRO results over time. The overall goal of an implementation framework is to help achieve “sustainable action” [45]. In the current context, numerous factors were perceived by staff as positive-influencing factors for the implementation and ongoing sustainability of this pathway in this clinical context.

Evaluating the process through the eyes of key stakeholders is essential in identifying strengths, limitations, and opportunities for improvement [40, 46]. A number of factors were identified as critical to the pathway’s success. Of these, the ‘intervention source’ was a key positive factor because the team had experienced the pathway structure as part of a prior research project [25]. Additionally, staff was aware of the evidence and necessity for coordinated care which is supported by National Comprehensive Cancer Network^®^ guidelines [47]. They were also aware of the evidence for proactive management of swallowing disorders [11, 13, 14, 20, 48]. Studies relating to prophylactic therapy outcomes published in subsequent years [15, 17, 19] continued to support the importance of sustaining the pathway. Through implementation of the research protocol, staff also had experience providing regular patient review/monitoring services and participated in regular collection of outcome data at specified time points. The time to successful implementation can be lessened when key stakeholders are involved in the process early on [49, 50]. Hence key positive factors adding support for establishing and sustaining the pathway were based on having had an ‘intervention source,’ which had been internally developed, strongly supported by evidence from multiple perspectives, and trialed by the MDT within the clinical context prior to implementation.

The functionality of supporting the pathway through the REDCap system enabled clinical and administrative staff to track patients easily and to monitor data collection of PROs and CRO, as well as adherence to appointments while the Patient Care Plan actively engaged patients in their care and program of appointments. The combined benefits of these ‘design quality and packaging’ elements would appear to be quantified in the current audit data, which revealed very few patients missed key appointments. However, while 40% of staff viewed the design elements of the pathway as positive factors to its success, 30% noted this as a barrier. This result may have reflected the fact that it is a complex pathway, with multiple systems and processes, and requires training requirements and time investment to use. Indeed 60% of the MDT members indicated that pathway ‘complexity’ was a barrier for both implementation and ongoing sustainability. Within this service, a minimum of 34 staff including physicians, allied health, research and administrative staff can be involved in the daily process. Treatment planning, scheduling multiple services at the same time, rescheduling for various reasons, performing interventions and treatments, entering data, designing templates and electronic medical record (EMR) platforms, educating patients and meeting to navigate patients are only some of the daily activities that are in constant motion. Coordination of appointments and follow-up with providers who have different schedulers in their departments further complicates workflow. Researchers have noted that any new care pathway must be adaptable and compatible with existing standard workflow processes, and with ongoing consideration of patient and staff demands on time and energy, to achieve successful integration into the MDT’s culture [39]. Hence ‘complexity’ is an issue which must be mitigated whenever possible.

In this service, only the construct of ‘patient needs’ within the CFIR domain *Outer Setting* was perceived to have an impact on the pathway, with most of the MDT identifying this as a positive-influencing factor to implementation and sustainability. Staff was invested in providing a service delivery model that would most effectively meet patient needs and reduce percieved gaps in service. Integral to the development of the D-HNCP was the desire to minimize patient burden as much as possible through coordinated appointments, to reduce the number of hospital visits, and to improve swallow function and nutritional intake. As evidenced by the audit data, patients on the pathway received regular routine care and were monitored closely for key outcomes. Overall, swallowing outcomes of the clinical cohort who received CRT under the pathway were comparable to those managed intensively during the earlier RCT indicating minimal long-term deficit, supporting multiple positive benefits for patients.

Of the CFIR domains, however, it was the *Inner Setting,* which appeared to have many of the main positive-influencing factors. Of these, the local ‘structural characteristics’ were particularly important. The D-HNCP was developed in a community hospital HNC center where support for HNC services is provided annually from a private endowment (Milton J Dance Endowment, https://www.gbmc.org/dance-story). The primary mission of the Dance Center is to provide MDT care for HNC patients. Hence, establishing an MDT model based on best practice health care for patients with HNC was positively aligned with the roots of the Dance Endowment’s mission. The pathway, therefore, matched the ‘culture’ of the organization and had ‘compatibility’ and ‘relative priority’ within the service. Having leaders within the Dance Center committed to the implementation ensured the endeavor was fully supported and funded. As such, ‘available resources’ and ‘leadership engagement’ were positive factors for implementation and sustainability. Damschroder and Hagedorn [51] emphasize the importance of having adequate funding before implementation to ensure that resources are not constrained by limitations in financial support. Because of this, engagement and input from key stakeholders are essential in prioritizing resources to achieve desired outcomes [51]. The lessons learned from the efficacy study [25] also provided the perfect ‘implementation climate,’ with staff ‘ready for an implementation’ to continue caring for HNC patients within an MDT structure, using routine outcome measurement and programmatic rigors.

Studies advocate for an MDT model where members communicate efficiently with each other to enhance coordination of care for patients [1, 3, 7]. To this end, the D-HNCP was also established with a clear ‘networks and communication’ structure, which staff viewed as a positive factor. Ensuring all patient data were available to all members via the REDCap platform was integral to ensuring open communication and sharing of information. Multiple strategies (weekly meetings, group emails, and documentation in the EMR and REDCap) were established to facilitate regular and clear communication, provide a positive ‘learning climate’ to discuss issues or barriers as they arose and ensure all staff had ‘access to knowledge and information.’ Participation from all members was supported with a high level of ‘leadership engagement’ which is essential for program sustainability [40, 51]. The leader of the D-HNCP’s responsibilities crossed clinical and administrative staff, which was helpful in problem-solving to implement and improve processes and support education and training for all team members.

Identifying external change agents, as well as clinical champions and opinion leaders to facilitate communication can assist in providing quality reflection and execution [51, 52]. The importance of having opinion leaders and champions supporting the D-HNCP implementation was recognized by most of the staff as critical to establishing and sustaining the pathway within the *Process* domain. The D-HNCP is a complex system requiring coordination and constant oversight to ensure adherence to all components of the pathway with ongoing leadership. Leaders and local champions were critical to ensuring disciplines worked together, respected individual roles and remained committed to the concept of providing care in the structure of an HNC care pathway. Acknowledging the many roadblocks and challenges of maintaining a complex pathway of patient care, these champions and leaders were integral to ensuring that the service was sustained and all patients received the best service. Within the pathway, a process of continuous monitoring and weekly feedback, as well as routine data audits by the Clinical Research Coordinator, was integral to help address any issues that arose. For example, the somewhat lower rates of PRO/CRO reporting noted with the 2013 group improved in 2014 group with more stringent monitoring, identification of D-HNCP champions, and ongoing staff education. Obtaining input from team members through ongoing reassessment positively influences acceptance of change and serves to identify areas for improvement in complex, multidisciplinary intervention-driven models [46, 51].

### Limitations

A major limitation of this study was the lack of data collected regarding patient perceptions of the pathway. While the high rates of attendance at appointments may suggest patients valued their care, future research into the patient’s perceptions and insights about the care model and ways to improve the care pathway for patients is needed. Another limitation was that a more formal and structured evaluation of staff perceptions of the implementation and sustainability of the D-HNCP was performed 5 years after its initiation in 2012. However,  continuous process improvement occurred over the 5 years through rigorous weekly input by the MDT, implementation issues raised, and MDT perceptions of barriers and facilitators were considered and modifications were implemented. A further limitation may be that the D-HNCP cohort swallowing outcomes data were compared to a prior research cohort instead of baseline data pre-implementation of the D-HNCP. Such a comparison may have been more informative regarding potential improvements in patient outcomes in a non-research setting. Furthermore, the audit data only examined the window of time up to 2-year post-admission. Ongoing auditing will be necessary to ensure that data integrity, adherence to pathway, and compliance of outcome data collection are maintained through the full 5 years of patient monitoring.

## Conclusions

Implementing and sustaining a multidisciplinary clinical pathway for HNC patients require significant time, staff resources, considerable financial investment, and perseverance. The current study highlights the range and complexity of influencing factors that need to be considered when planning clinical pathways for HNC care in other services. This study demonstrated that once established, multiple communication and monitoring processes, as well as leadership and team commitment, are needed to yield success and ensure sustainability. The process of implementation has multiple phases requiring a level of engagement and commitment by the whole MDT, including ongoing team problem-solving and process improvement. Despite the complexity, positive clinical, patient, and service outcomes can be achieved through implementation of a well-designed care pathway.
